# Pharmaceutical quality and cost-effectiveness of branded versus generic medicines across 22 drug types and 8 therapeutic categories: a citizen-funded comparative study from regulated retail outlets in Kerala, India

**DOI:** 10.3389/fphar.2026.1799574

**Published:** 2026-06-03

**Authors:** Cyriac Abby Philips, Arif Hussain Theruvath, Aryalakshmi Sreemohan, Ambily Baby, Shinsmon Jose, Mathew Philips, Tony Philip, Chandrasekhar Ramesh

**Affiliations:** 1 Clinical and Translational Hepatology, The Liver Institute, Center for Excellence in Gastrointestinal Sciences, Rajagiri Hospital, Aluva, Kerala, India; 2 Department of Clinical Research, The Liver Institute, Center for Excellence in Gastrointestinal Sciences, Rajagiri Hospital, Aluva, Kerala, India; 3 Division of Infectious Diseases, College of Medicine, University of Cincinnati, Cincinnati, OH, United States; 4 Department of Orthopaedic Surgery, Abeer Medical Group, Jeddah, Saudi Arabia; 5 Department Surgical Dentistry, Al Ittihad Polyclinic and Diagnostic Centre, Ajman, United Arab Emirates; 6 Mission for Ethics and Science in Healthcare (MESH), Thiruvananthapuram, Kerala, India

**Keywords:** bioequivalance, generics, low cost, pharmaceutical, quality control

## Abstract

**Background:**

Despite India’s status as a global pharmaceutical hub, skepticism regarding the quality of generic medicines persists among healthcare professionals and the public. This study aimed to comprehensively evaluate the pharmaceutical quality and cost-effectiveness of branded versus generic medicines to address these concerns and inform healthcare policy.

**Methods:**

In a cross-sectional analytical study, 131 samples of 22 essential medicines across 8 therapeutic categories were procured using convenience sampling from 7 diverse source categories of licensed retail outlets in Kerala, India, including government, private generic, and branded outlets. Samples underwent rigorous testing for description, dosage uniformity, dissolution, impurities, and assay content in an accredited laboratory according to Indian Pharmacopoeia 2022 standards. Cost-effectiveness was analysed using unit pricing and price ratios.

**Results:**

All 131 samples (100%) met all pharmacopeial standards, demonstrating pharmaceutical equivalence across all quality parameters, regardless of whether they were generic, brand, or priced differently. Generic medicines were, on average, 48.6% cheaper than branded equivalents. The study revealed substantial price disparities, with the most expensive branded products costing up to 13.9 times as much as the lowest-priced generics. Government-run Jan Aushadhi outlets consistently offered the most affordable options across 81.8% of medicine categories tested in the study.

**Conclusion:**

The data confirm that low-cost government generic programs maintain rigorous quality control comparable to that of expensive private brands, highlighting a significant opportunity for cost savings in chronic disease management. Generic medicines tested in this study are pharmaceutically equivalent to their branded counterparts. Shifting to quality-assured generics, particularly through effective government retail schemes, will substantially reduce out-of-pocket healthcare expenditure and improve treatment adherence without compromising therapeutic quality.

## Introduction

### Background and rationale

Access to quality-assured medicines is fundamental to achieving positive health outcomes globally. The global burden of pharmaceutical expenditure poses a significant challenge to healthcare systems worldwide, particularly in low- and middle-income countries (LMICs) where healthcare financing remains predominantly out-of-pocket. Generic medicines, which are bioequivalent to their branded counterparts, play a crucial role in improving treatment adherence and outcomes, particularly in resource-limited settings (Ozawa et al., 2020). The pharmaceutical industry in India is among the largest globally, supplying approximately 20% of the world’s generic medicines (Joshi et al., 2019). Despite this, concerns about the quality of generic medicines relative to branded products persist among healthcare professionals and the public (Hussar, 2020).

The World Health Organization (WHO) estimates that substandard and falsified medicines account for approximately 10% of medicines in low- and middle-income countries, with associated annual costs of approximately US$31 billion (World Health Organization, 2017). Such findings have contributed to skepticism about the quality of generic medicines. Studies have demonstrated that healthcare professionals, including physicians, often harbour negative perceptions of generic medicines, viewing them as lower quality or associated with more side effects than branded alternatives (Colgan et al., 2015; Priyadarsini et al., 2023). These perceptions may lead to preferential prescribing of more expensive branded medicines, thereby limiting access to healthcare for economically disadvantaged populations. Quality assessment of pharmaceutical products typically involves evaluation of multiple parameters including identification tests, weight uniformity, dissolution testing, impurity profiling, and assay of active pharmaceutical ingredients (Yang et al., 2025). The Indian Pharmacopeia (IP) provides comprehensive specifications for these parameters, ensuring that medicines marketed in India meet established safety and efficacy standards.

Previous studies comparing the quality of branded and generic medicines have yielded variable results. A comprehensive study of angiotensin receptor blockers demonstrated that both branded and generic formulations met established pharmacopeial standards for impurities, active drug content, and dissolution (Sharma et al., 2024). Similarly, a study on antiretroviral medications in Asian settings found that all sampled generic products met International Pharmacopeia standards (Sapsirisavat et al., 2016). However, other studies have reported quality failures in generic products, particularly in settings with limited regulatory oversight (Sardella et al., 2021; Ravinetto et al., 2018). India, despite being recognized as the “pharmacy of the world” and a leading manufacturer of generic medicines, continues to grapple with paradoxical challenges in ensuring universal access to quality, affordable medicines for its population of over 1.4 billion. Medicines account for approximately 62%–69% of household out-of-pocket spending on healthcare in India, creating substantial economic vulnerability and pushing millions below the poverty line annually (Sriram and Albadrani, 2022; Kamath et al., 2024).

### Gaps in literature and significance of study

The quality of generic medicines is critical for public health, yet the existing evidence base remains fragmented and methodologically limited. A systematic review and meta-analysis estimated that 13.6% of medicines in low- and middle-income countries (LMICs) are of poor quality, with higher prevalence reported in Africa (18.7%) and Asia (13.7) (Ozawa et al., 2018). While concerning, these estimates obscure substantial heterogeneity in study design, sampling strategies, and medicine selection. Many studies rely on convenience sampling and focus on a narrow range of products.

Most published investigations have concentrated on specific therapeutic classes, particularly antimalarials and antibiotics, rather than medicines used for chronic disease management. This limits the generalizability of findings and reduces their relevance for long-term population health. In addition, methodological weaknesses increase the risk of systematic bias and restrict meaningful comparisons across studies. Importantly, most studies have evaluated generic medicines in isolation or assessed them solely against pharmacopeial standards. Few have simultaneously tested branded and generic products sourced from the same market. As a result, the question of whether commonly available generic medicines perform equivalently to branded alternatives remains inadequately addressed.

Evidence on cost-effectiveness is also sparse. For example, A systematic review of cardiovascular medicine quality found a failure frequency of 15.4% among 3,414 samples tested but noted that the majority of samples came from LMICs and that the relationship between quality and price was not systematically examined (Do et al., 2021). Similarly, although public-sector generic medicine programs have expanded in India, peer-reviewed data on the quality of medicines distributed through these channels remain scarce. Studies from Africa and Asia suggest that government-regulated supply chains are underrepresented in quality assessments, with most samples drawn from private or wholesale markets (Asrade Mekonnen et al., 2024). Furthermore, many studies sample medicines from hospital pharmacies or wholesale distributors, which may not reflect products accessible to patients through retail outlets. Quality assessments are most informative when they represent medicines available at the point of care, where purchasing decisions are made.

To address these gaps, we adopted a pragmatic and comprehensive study design. We evaluated medications across 22 therapeutic categories, including cardiovascular, metabolic, gastrointestinal, respiratory, and infectious diseases. Second, both branded and generic products were procured from the same geographic market to allow direct comparison. Third, we tried to integrate quality assessment with detailed cost-effectiveness analysis, providing actionable information on value for money across procurement sources. Fourth, medicines from government-sponsored generic programs were evaluated alongside private-sector products. Together, this approach enables a more representative assessment of the quality and cost-effectiveness of essential medicines across commonly used therapeutic classes using standardized pharmacopeial methods.

## Methods

### Study design

This was a cross-sectional analytical study conducted as part of the MESH (Mission for Ethics and Science in Healthcare) Citizens Generic vs. Branded Drugs Quality Project. MESH, a not-for-profit Registered Society is a collective of national and international experts, united together with a single primary objective: to assist public and government so that all Indian citizens can have access to affordable evidence-based medicines/interventions, in an ethical manner. The study was designed to assess the pharmaceutical quality and cost-effectiveness of medicines available through multiple procurement channels in Kerala, India. The study was conducted between August 2025 and December 2025 and did not involve human subjects; therefore, ethical approval was not required. The study design and reporting followed the MEDQUARG (Medicine Quality Assessment Reporting Guidelines) framework for pharmaceutical quality studies.

### Sample selection and procurement

The sampling frame comprised solid oral dosage forms (tablets and capsules) of medicines commonly prescribed for both acute and chronic conditions in India. Therapeutic categories were selected based on: (1) prevalence of use in the Indian population, (2) availability across multiple procurement sources, (3) representation of different pharmacological classes, and (4) relevance to both commonly encountered communicable and non-communicable disease management. Medicines were procured from seven distinct sources representing the spectrum of pharmaceutical supply chains available to consumers.

1. Jan Aushadhi (JAUSH): Central government-operated generic medicine outlets, providing quality-assured generic medicines at controlled prices.
2. Kerala Medical Services Corporation Limited (KMSCL): State government corporation responsible for centralized procurement and supply of essential medicines to public health facilities.
3. DAVA India and 4. Generic Aadhar (GENADH): Both private pharmacy network providing generic medicines at competitive prices.
4. Generic distributors (GENERIC): Independent pharmaceutical distributors supplying local/trade unbranded generic medicines.
5. Branded generic manufacturers (BGENERIC): Products marketed under manufacturer brand names but positioned as generic alternatives.
6. Branded medicine manufacturers (BRAND): Original branded products from established pharmaceutical companies.

Samples were collected using a structured procurement protocol. Within each therapeutic category, specific molecules were selected based on the following criteria: (1) inclusion in the National List of Essential Medicines (NLEM) of India or wide prevalence of clinical use, (2) availability in solid oral dosage form (tablet or capsule), (3) availability from at least two of the seven source categories to enable cross-source comparison, and (4) existence of Indian Pharmacopoeia 2022 monograph specifications (or approved alternatives) for quality testing. For each therapeutic category, attempts were made to procure products from all available sources. Procurement was conducted by trained field staff who purchased medicines as regular consumers (mystery shopping approach) to ensure samples reflected actual market availability. Sampling was conducted using a pragmatic convenience approach rather than probability-based sampling. No formal randomization or stratification by geography or outlet was employed. The selection therefore reflects products available through regulated retail channels at the time of procurement and may not represent products available through unlicensed or informal markets, or the complete range of products available across all licensed outlets in the state or country. All samples were purchased from licensed retail outlets with valid permits. Sample details including batch number, manufacturing date, expiry date, manufacturer name and address, maximum retail price (MRP), and pack size were recorded at the time of procurement.

Convenience sampling was chosen as the pragmatic approach for this citizen-funded study because probability-based sampling of all licensed pharmaceutical outlets across Kerala was not feasible given logistical and financial constraints. This approach is consistent with the majority of post-market pharmaceutical quality surveillance studies, where convenience or purposive sampling remains the predominant methodology. Nonetheless, by procuring samples from seven distinct source categories spanning government, private generic, and branded outlets, we aimed to capture the breadth of regulated procurement channels available to consumers, even though formal representativeness cannot be claimed.

Inclusion criteria included: (1) Solid oral dosage forms (tablets or capsules), (2) Within labelled shelf life at time of procurement and anticipated testing, (3) Intact primary packaging, (4) Clearly legible labelling with required regulatory information; while exclusion criteria included: (1) Damaged or compromised packaging, (2) Missing or illegible labelling, and (3) Products within 3 months of expiry to ensure adequate shelf life for testing. Procured samples were stored in their original packaging under controlled conditions until transfer to the testing laboratory. All samples were assigned unique alphanumeric codes prior to submission for testing. Laboratory analysts performing quality assessments were blinded to sample source category (government, private generic, or branded) and specific brand identity throughout the testing process. Data analysis personnel subsequently interpreted results using only coded identifiers, with unblinding occurring only after completion of all analyses. A chain of custody documentation was maintained throughout the process, recording sample identifiers, collection dates, storage conditions, and transfer dates.

### Quality assessment methods

All quality testing was performed by Eureka Analytical Services (https://eurekaserv.com/about-us/), a National Accreditation Board for Testing and Calibration Laboratories (NABL) India, and the United States – Food and Drug Administration approved ISO/IEC 17025 accredited pharmaceutical testing laboratory, between December 12-22, 2025. The laboratory maintains Good Laboratory Practice (GLP) compliance and is approved by the Central as well as State Drug Control Authority for pharmaceutical quality analysis. All quality parameters were assessed according to Indian Pharmacopoeia 2022 (IP-2022) monograph specifications (Indian Pharmacopoeia Commission, 2022). Additionally, Rifaximin was tested using IP Addendum 2024 specifications, while Febuxostat was tested using in-house specification based on the Japanese Pharmacopeia standards, because IP monograph currently does not exist for this drug. Certified reference standards traceable to national/international pharmacopeial standards were used for all assays.

### Quality parameters assessed

#### Five quality parameters were systematically assessed for all samples

*Description and physical characteristics*: Visual inspection of dosage forms for colour, shape, size, surface characteristics, and physical integrity. Tablets were examined for capping, lamination, chipping, and discoloration. Capsules were examined for shell integrity, proper filling, and evidence of moisture damage.

*Uniformity of dosage*: Uniformity of dosage units was evaluated using the appropriate method based on drug content and dosage form. Weight uniformity testing was performed on tablets and capsules containing ≥25 mg of active ingredient or where the active ingredient comprises ≥25% of the dosage unit weight, on film-coated tablets or hard gelatin capsules. Twenty units are individually weighed and the percentage deviation from mean weight is calculated, with acceptance limits of ±5.0% for tablets weighing ≥250 mg and ±7.5% for smaller tablets and capsules. Content uniformity testing was performed on formulations containing <25 mg of active ingredient or where the drug comprises <25% of the dosage unit weight and in case of coated tablets (other than film-coated) and soft gelatin capsules. Ten individual units are assayed separately, and the acceptance value (AV) is calculated, with a specification of AV ≤15.0 (equivalent to 85.0%–115.0% of label claim). For example, metformin 500 mg or paracetamol 650 mg would undergo weight uniformity testing due to their high dose, while folic acid or prednisolone 10 mg would undergo content uniformity testing due to their inherent low dose.

*Dissolution testing*: Evaluation of drug release characteristics was performed using USP dissolution apparatus. Testing parameters included dissolution medium, medium volume, apparatus rotation speed and sampling time points per monograph requirements. For gastro-resistant and modified-release formulations, two- or three-point dissolution testing was performed as per specifications.

*Related substances/impurities*: Chromatographic analysis to identify and quantify degradation products, process impurities, and other related substances. High-performance liquid chromatography (HPLC) with UV detection was used following monograph-specified conditions including column specifications, mobile phase composition, flow rate, detection wavelength, and injection volume. Individual and total impurity limits were applied as specified in respective monographs (typically ≤0.1–0.5% for individual impurities and ≤1.0–2.0% for total impurities).

*Assay (Active Pharmaceutical Ingredient, API – Content)*: Quantitative determination of API content using validated HPLC methods specified in IP-2022 monographs. Sample preparation, chromatographic conditions, and calculation methods followed monograph specifications. Results were expressed as percentage of label claim. Acceptance criteria: 90.0%–110.0% of label claim (or as specified in individual monographs, with some permitting 95.0%–105.0% for narrow therapeutic index drugs). Identity confirmation was inherent to the HPLC-based assay methodology employed. Compliant assay results required retention time correspondence with certified reference standards under specified chromatographic conditions, thereby confirming the identity of the active pharmaceutical ingredient and excluding incorrect or substituted substances.

All analytical methods were validated for specificity, linearity, accuracy, precision, and robustness prior to sample analysis. System suitability criteria were verified before each analytical run. Positive controls (certified reference standards) and negative controls (blanks) were included in each analytical batch. Repetition of investigations were conducted according to laboratory standard operating protocols when initial results fell outside acceptance limits, including repeat testing from original sample preparation and re-preparation from new sample aliquots.

### Cost-effectiveness analysis

Maximum retail price (MRP) was recorded from product packaging at the time of procurement. MRP in India includes all taxes and represents the maximum legal price at which products can be sold to consumers. Pack sizes were recorded to calculate unit prices. Unit price was calculated as: unit price (₹) = MRP (₹) of the pack ÷ number of units per pack. This standardized metric enables direct comparison across products with different pack sizes. The following cost-effectiveness metrics were calculated.

- Price ratio – Ratio of highest to lowest unit price within each therapeutic category, calculated as: Price ratio = highest unit price ÷ lowest unit price. This metric quantifies the magnitude of price variation available to consumers.
- Potential daily savings – Difference between highest and lowest unit prices within each category: Daily savings (₹) = highest unit price – lowest unit price.
- Potential monthly or annual savings – Projected savings assuming one unit per day consumption: Month-based savings (₹) = daily savings × months (in days). This conservative estimate is relevant for chronic disease medications requiring daily dosing. Table 1 summarizes the quality parameters assessed, testing methods, and acceptance criteria applied.

**TABLE 1:** Summary of quality parameters and acceptance criteria.

Parameter	Test method	Acceptance criteria	References
Description	Visual inspection	Compliance with monograph description	IP-2022
Uniformity of dosage units	Weight variation (tablets ≥250 mg, capsules) or content uniformity (<25 mg API)	Weight: ±5.0% (tablets ≥250 mg), ±7.5% (smaller units); content: AV ≤15.0	IP-2022
Dissolution	USP apparatus per monograph	Q ≥ 70% (gastro-resistant), ≥75–80% (immediate-release, drug-specific)	IP-2022 or IP-2024 addendum or appropriate, approved in-house validated method
Disintegration	USP apparatus (highly soluble drugs)	≤30 min (tablets), ≤3 min (dispersible)	IP-2022
Related substances	HPLC per monograph	Individual: ≤0.1–0.5%; total: ≤1.0–2.0% (monograph-specific)	IP-2022
Assay	HPLC per monograph	90.0%–110.0% of label claim (or monograph-specific)	IP-2022

IP-2022, Indian Pharmacopoeia 2022; API, active pharmaceutical ingredient; AV, acceptance value.

### Statistical analysis

The primary analysis was descriptive in nature. Quality outcomes were summarized as compliance frequencies and percentages. Continuous quality parameters (dissolution values, assay percentages) were summarized using means, standard deviations, medians, and ranges. Cost metrics were similarly summarized using descriptive statistics. Given the convenience sampling design and the descriptive intent of the study, formal inferential statistical comparisons between source categories were not performed; observed differences should be interpreted as descriptive observations rather than statistically confirmed effects. Coefficients of variation (CV) were calculated to characterize dispersion within groups. For cost-effectiveness analyses, Maximum Retail Price (MRP) was used as the standardized price metric; samples with ₹0.00 unit price were excluded from mean and median price calculations but reported separately. All analyses were performed using Microsoft Excel (Microsoft Corporation, Redmond, WA) and Python 3.9.0 with *pandas* and *numpy* libraries.

### Ethical considerations

This study involved analysis of commercially available pharmaceutical products and did not involve human participants, human biological samples, or access to identifiable personal data. Therefore, institutional ethics committee review was not required. All medicines were procured through legitimate retail channels at market prices. The study was conducted in accordance with the principles of research integrity and transparent reporting.

## Results

**FIGURE 1:**
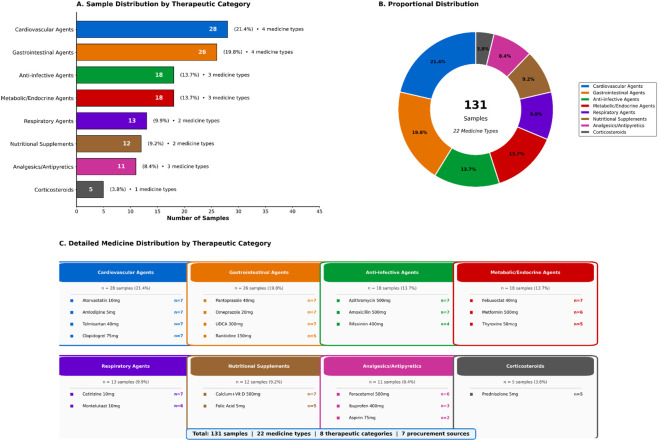
Sample distribution by therapeutic category – Distribution of 131 medicine samples across 8 therapeutic categories and 22 medicine types from 7 procurement sources. **(A)** Sample distribution by therapeutic category showing number of samples and percentage contribution. **(B)** Proportional distribution displayed as a donut chart. **(C)** Detailed breakdown of individual medicines within each therapeutic category, showing specific formulations and sample sizes.

A total of 131 medicine samples were collected and analysed, representing 22 medicines from 8 therapeutic categories from seven procurement sources. The analysed drugs included (Figure 1 and Supplementary Document 1).

- *Analgesics/anti-inflammatory*: acetaminophen (Paracetamol) 500 mg, Ibuprofen 400mg, Prednisolone 10 mg
- *Antibiotics*: Azithromycin 500 mg, Amoxicillin 500mg, Rifaximin 400 mg
- *Gastrointestinal agents*: Ranitidine 150mg, Pantoprazole 40mg, Omeprazole 20mg, Ursodeoxycholic Acid (UDCA) 300 mg
- *Cardiovascular drugs*: Atorvastatin 10mg, Amlodipine 5 mg, Telmisartan 40mg, Clopidogrel 75 mg, Aspirin 75 mg
- *Respiratory and anti-allergy medications*: Montelukast 10mg, Cetirizine 10 mg
- *Metabolic/endocrine agents*: Metformin 500mg, Levothyroxine (Thyroxine Sodium) 50 mcg, Febuxostat 40 mg
- *Nutritional supplements*: Calcium with Vitamin D 500 mg and Folic acid

The distribution by source was: Branded medicines (BRAND) n = 41 (31.3%), Branded generics (BGENERIC) n = 32 (24.4%), Jan Aushadhi (Central Government generics program, JAUSH) n = 21 (16.0%), Dava India (private limited generics program, DAVA) n = 14 (10.7%), Generic distributors (GENERIC) n = 11 (8.4%), Generics Aadhar (private limited, CSR funded generics program, GENADH) n = 9 (6.9%), and Kerala Medical Services Corporation (State government generics program, KMSCL) n = 3 (2.3%). All samples were within their labelled shelf life, with expiry dates ranging from June 2026 to December 2028.

### Dissolution results

**FIGURE 2:**
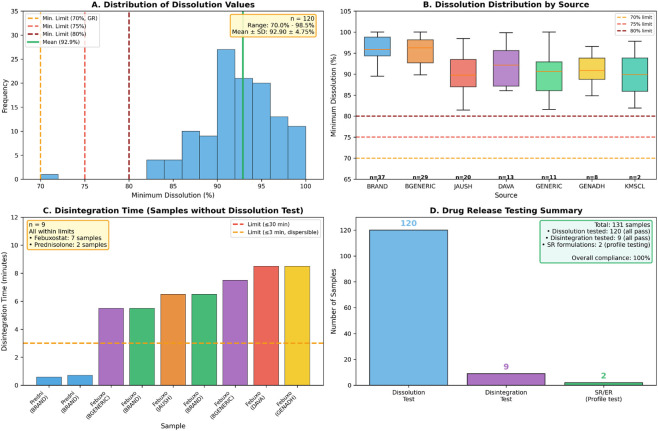
Drug release testing results for 131 drug samples - **(A)** Distribution of minimum dissolution values for samples undergoing standard single-point dissolution testing (n = 120), with pharmacopeial threshold limits indicated by dashed lines (70% for gastro-resistant formulations, 75% for most immediate-release tablets, 80% for certain antibiotics). Green solid line indicates mean dissolution (92.90%). **(B)** Box plots showing dissolution distribution stratified by procurement source. Sample sizes indicated below each box. **(C)** Disintegration times for samples tested per IP-2022 monograph specifications for highly soluble drugs: Febuxostat tablets (n = 7; limit ≤30 min) and Prednisolone dispersible tablets (n = 2; limit ≤3 min). **(D)** Summary of drug release testing methods applied across all 131 samples, demonstrating 100% pharmacopeial compliance. SR/ER, sustained-release/extended-release formulations (Metformin) requiring multi-timepoint dissolution profile testing.

Among the 131 samples, drug release characteristics were assessed using the appropriate method per IP-2022 monograph requirements (Figure 2). A total of 120 samples underwent standard single-point dissolution testing with a mean minimum dissolution of 92.90% (SD: 4.75%, range: 70.0%–98.5%). Two Metformin sustained/extended-release formulations required dissolution profile testing per IP-2022 requirements for modified-release products; both demonstrated compliant drug release across multiple time points. All samples exceeded their respective pharmacopeial dissolution thresholds (≥70% for gastro-resistant formulations, ≥75% for most immediate-release tablets, ≥80% for certain antibiotics). Nine samples (7 Febuxostat and 2 Prednisolone formulations) underwent disintegration testing instead of dissolution, as specified by their IP-2022 monographs for solid oral dosage forms containing highly soluble drugs. Febuxostat samples showed disintegration times of 5–9 min (limit: ≤30 min), while Prednisolone disintegrated within 30–50 s (limit: ≤3 min), all well within accepted standards.

### Assay results

**FIGURE 3:**
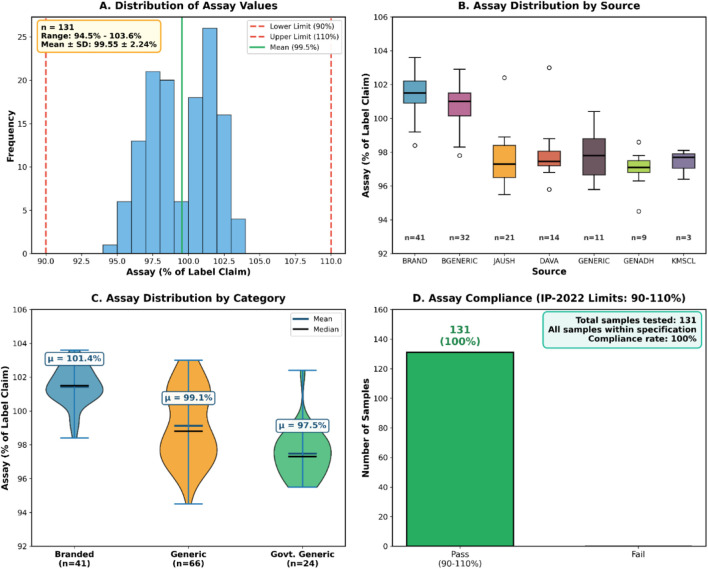
Assay (drug content) analysis results – Assay results for all 131 medicine samples tested according to Indian Pharmacopoeia 2022 limits (90%–110% of label claim). **(A)** Distribution of assay values showing range from 94.5% to 103.6% with mean ± SD of 99.55% ± 2.24%. The histogram demonstrates that all values fall well within pharmacopeial limits, with the majority clustering between 97% and 103%. **(B)** Box plot comparison of assay values by procurement source. Sample sizes: BRAND (n = 41), BGENERIC (n = 32), JAUSH (n = 21), DAVA (n = 14), GENERIC (n = 11), GENADH (n = 9), KMSCL (n = 3). Branded products showed the highest mean assay (101.44%), while government generic sources (JAUSH, KMSCL) showed slightly lower but fully compliant means (97.50% and 97.40%, respectively). **(C)** Violin plot showing assay distribution by broad category: Branded (n = 41, μ = 101.4%), Generic (n = 66, μ = 99.1%), and Government Generic (n = 24, μ = 97.5%). All categories demonstrated tight clustering within specifications. **(D)** Compliance summary showing all 131 samples (100%) passed assay testing within the 90%–110% specification range.

All 131 samples (100%) underwent quantitative assay testing (Figure 3), with a mean assay value of 99.55% of label claim (SD: 2.24%, range: 94.5%–103.6%). By source category: branded medicines showed the highest mean assay of 101.44% (SD: 1.08%), branded generics 100.76% (SD: 1.40%), DAVA pharmacy chain 97.84% (SD: 1.65%), independent generics 97.76% (SD: 1.43%), Jan Aushadhi 97.50% (SD: 1.57%), KMSCL 97.40% (SD: 0.89%), and GENADH 96.99% (SD: 1.13%). All values were well within the pharmacopeial acceptance range of 90%–110%. The lowest assay value (94.5%) was observed in an Omeprazole capsule from the GENADH source, which still met the specification of 90.0%–110.0% for this product. The highest assay value (103.6%) was found in a Folic Acid tablet from a branded source. The coefficient of variation was low across all categories (≤2.25%), indicating consistent manufacturing quality regardless of source. Branded products showed lower coefficients of variation in assay values (CV: 1.07%) compared to generic alternatives (CV: 2.16%) and government generics (CV: 1.52%), though all individual values across all source categories were well within pharmacopeial acceptance limits.

### Uniformity of dosage units

**FIGURE 4:**
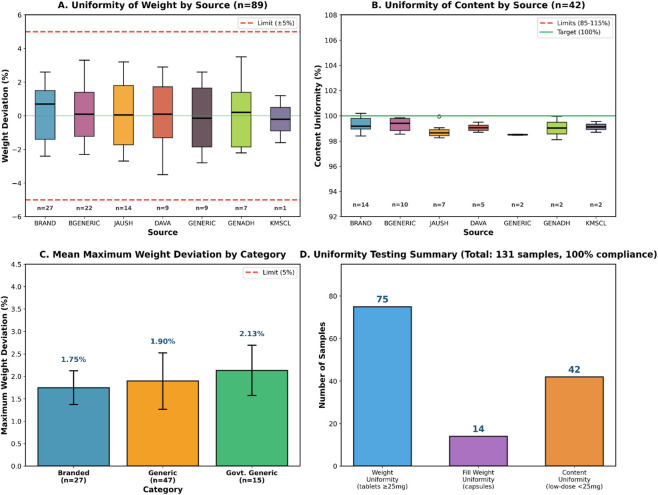
Uniformity testing results – Uniformity of dosage units testing results for 131 samples. **(A)** Box plot of weight uniformity (deviation from average weight) by procurement source for 89 samples tested by weight variation method. Sample sizes: BRAND (n = 27), BGENERIC (n = 22), JAUSH (n = 14), DAVA (n = 9), GENERIC (n = 9), GENADH (n = 7), KMSCL (n = 1). All samples fell within ±5% pharmacopeial limits (indicated by red dashed lines). **(B)** Box plot of content uniformity by procurement source for 42 samples tested by content uniformity method. Sample sizes: BRAND (n = 14), BGENERIC (n = 10), JAUSH (n = 7), DAVA (n = 5), GENERIC (n = 2), GENADH (n = 2), KMSCL (n = 2). All values fell within the 85%–115% acceptance criteria, with means ranging from 98.1% to 100.2%. **(C)** Mean maximum weight deviation by broad category: Branded (n = 27, 1.75% ± 0.38%), Generic (n = 47, 1.90% ± 0.64%), and Government Generic (n = 15, 2.13% ± 0.58%). Error bars represent standard deviation. All categories remained well below the 5% limit. **(D)** Uniformity testing summary: 75 tablet samples underwent weight uniformity testing, 14 capsule samples underwent fill weight uniformity testing, and 42 low-dose (<25 mg) formulations underwent content uniformity testing. Total: 131 samples, 100% compliance.

Uniformity testing (Figure 4) was performed on all 131 samples using the appropriate method based on dosage form and drug content per IP-2022 guidelines. Weight uniformity testing was conducted on 89 samples (tablets ≥250 mg and uncoated tablets), with a mean maximum deviation of 1.89% (SD: 0.57%, range: −3.5% to +3.5%), well within the ±5.0% acceptance criterion for tablets and ±7.5% for capsules. Content uniformity testing was performed on 42 samples containing low-dose active ingredients (<25 mg per unit), with a mean content uniformity of 99.14% (SD: 0.58%, range: 93.4%–103.8%), meeting the acceptance criterion of 85.0%–115.0%. By category, branded products showed mean maximum weight deviation of 1.75% (SD: 0.38%), generic products 1.90% (SD: 0.64%), and government generics 2.13% (SD: 0.58%). The tight distribution across all sources indicates consistent manufacturing processes, with government generic programs demonstrating comparable quality control standards to commercial alternatives.

### Related substances/Impurities

Out of 131 samples, 94 samples underwent tests for related substance and in 37 samples, as per IP, this parameter was not a regulatory requirement. Nonetheless, general impurity and heavy metals analysis were performed on all samples. No sample exceeded 80% of its applicable individual or total impurity specification limit. No samples showed chromatographic evidence of excessive degradation products or process impurity or general contamination. Physical description assessment confirmed that all samples met their respective monograph descriptions for colour, shape, size, and surface characteristics, with no observations of capping, lamination, chipping, discoloration, or moisture damage. This finding is particularly significant given concerns sometimes raised about manufacturing quality in generic production.

In summary, all 131 samples (100%) achieved full compliance with Indian Pharmacopoeia 2022 standards across all five quality parameters assessed. No failures were recorded for description, uniformity of dosage units, dissolution, related substances, or assay tests between generic or their branded counterparts. This complete pass rate was consistent across all seven procurement sources and all 22 therapeutic categories.

### Cost-effectiveness analysis results

**FIGURE 5:**
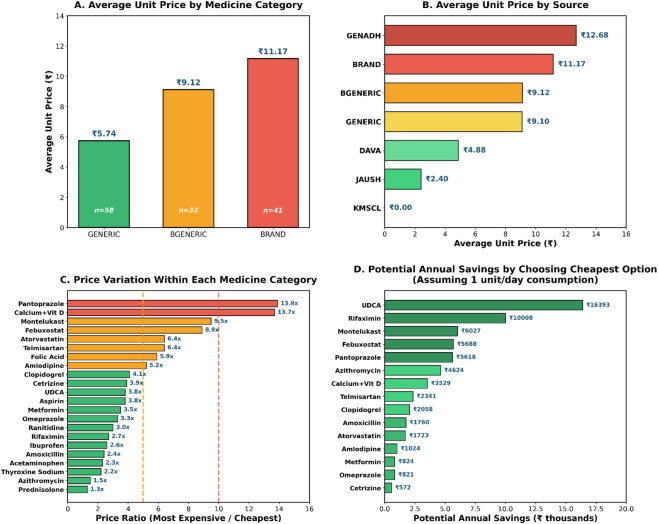
Price analysis overview – Price analysis of 131 medicine samples across procurement sources and categories. **(A)** Average unit price by medicine category: Generic (n = 58, ₹5.74), Branded Generic (n = 32, ₹9.12), and Brand (n = 41, ₹11.17). Branded products cost 95% more than generics on average. **(B)** Average unit price by procurement source in descending order: GENADH (₹12.68), BRAND (₹11.17), BGENERIC (₹9.12), GENERIC (₹9.10), DAVA (₹4.88), JAUSH (₹2.40), and KMSCL (₹0.00, provided free). Jan Aushadhi (JAUSH) offered the lowest-cost commercially available option. **(C)** Price variation within each of 22 medicine categories, expressed as price ratio (most expensive ÷ cheapest). Pantoprazole showed the highest variation (13.9×), followed by Calcium + Vitamin D (13.7×) and Montelukast (9.5×). Vertical dashed lines indicate 5× and 10× price ratios. **(D)** Potential annual savings by choosing the cheapest quality-tested option (assuming 1 unit/day consumption). UDCA offered maximum savings (₹16,393/year), followed by Rifaximin (₹10,008/year) and Montelukast (₹6,027/year).

In the context of equal pharmaceutical quality, substantial price variations were observed across medicine categories. Generic medicines (n = 58) had a mean unit price of ₹5.74 (median: ₹2.90, range: ₹0.00-₹38.72). Branded generics (n = 32) averaged ₹9.12 (median: ₹6.50, range: ₹1.21-₹39.80). Branded medicines (n = 41) averaged ₹11.17 (median: ₹8.40, range: ₹0.81-₹61.60). Overall, generic medicines were 48.6% cheaper than branded medicines and 37.1% cheaper than branded generics (Figure 5).

**FIGURE 6:**
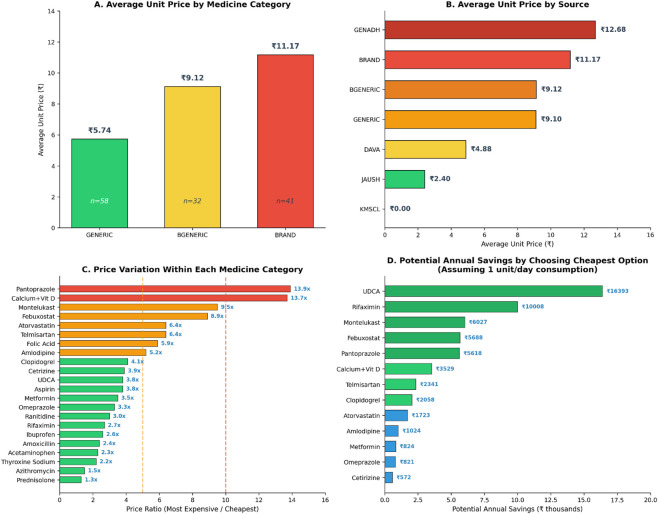
Price analysis of generic and branded medicines – **(A)** Average unit price comparison across medicine categories: unbranded generic (GENERIC), branded generic (BGENERIC), and innovator brand (BRAND). Sample sizes indicated within bars. **(B)** Average unit price by procurement source, ranked from lowest to highest. KMSCL, Kerala Medical Services Corporation Limited; JAUSH, Jan Aushadhi; DAVA, state drug distribution outlets. **(C)** Price variation within each medicine category, expressed as the ratio of most expensive to cheapest available option. Dashed lines indicate 5× and 10× price differential thresholds. **(D)** Potential annual savings (₹) achievable by selecting the lowest-cost option for each medicine, assuming once-daily consumption. UDCA, ursodeoxycholic acid.

Government-supported outlets demonstrated the lowest prices. Kerala Medical Services Corporation (KMSCL) provided three medicines (amlodipine, metformin, thyroxine sodium) at zero cost through public distribution. Jan Aushadhi had a mean unit price of ₹2.40 (median: ₹0.90). DAVA India followed at ₹4.88 mean unit price. In contrast, branded medicines averaged ₹11.17 and Generic Aadhar outlets averaged ₹12.68 – notably higher than other generic sources despite “generic” branding (Figure 6).

**FIGURE 7:**
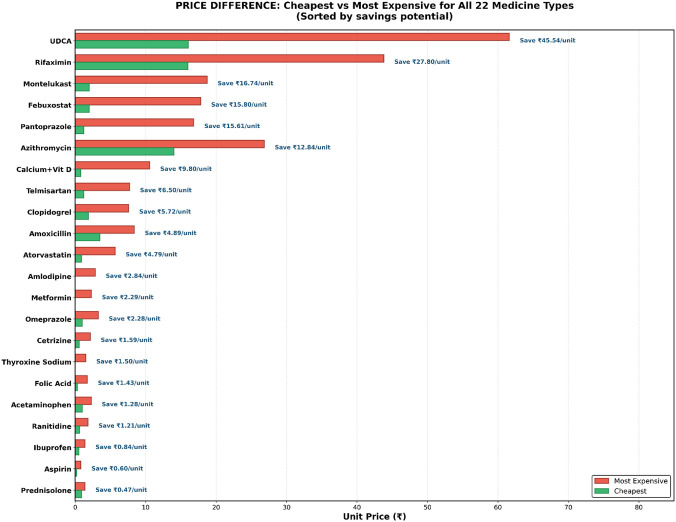
Price difference analysis and savings potential for all medicines – Comprehensive price difference analysis for all 22 medicine types, sorted by savings potential from highest to lowest. Paired horizontal bars show cheapest (green) and most expensive (red) unit prices for each medicine, with calculated savings per unit displayed. Top savings opportunities: UDCA (Save ₹45.54/unit), Rifaximin (Save ₹27.80/unit), Montelukast (Save ₹16.74/unit), Febuxostat (Save ₹15.80/unit), and Pantoprazole (Save ₹15.61/unit). Moderate savings (₹5–15/unit): Azithromycin (₹12.84), Calcium + Vitamin D (₹9.80), Telmisartan (₹6.50), Clopidogrel (₹5.72). Lower savings (<₹5/unit): Amoxicillin (₹4.89), Atorvastatin (₹4.79), Amlodipine (₹2.84), Metformin (₹2.29), Omeprazole (₹2.28), Cetirizine (₹1.59), Thyroxine Sodium (₹1.50), Folic Acid (₹1.43), Acetaminophen (₹1.28), Ranitidine (₹1.21), Ibuprofen (₹0.84), Aspirin (₹0.60), and Prednisolone (₹0.47). All price comparisons are between quality-tested samples that met pharmacopeial specifications.

Jan Aushadhi was the cheapest source for 18 out of 22 (81.8%) medicine types, KMSCL for 3 (13.6%), and DAVA India for 1 (4.5%). No branded or branded generic product was the cheapest option in any category. Conversely, branded medicines were the most expensive option for 16/22 (72.7%) categories, and branded generics for 5/22 (22.7%). One branded generic product was unexpectedly the costliest in its category at ₹16.82/unit. The mean price ratio across all 22 categories was 5.0x (range: 1.5x-13.9x), indicating that on average, the most expensive option cost five times more than the cheapest. Categories with highest price ratios (greatest potential savings) included: Pantoprazole 40 mg (13.9×), Calcium + Vitamin D (13.7×), Montelukast 10 mg (9.5×), and Febuxostat 40 mg (8.9×) (Figure 7). For example, the medicines with the highest individual savings potential were.

- UDCA: ₹16,621/year savings (UDCA-300 from JAUSH at ₹16.06 vs. Udiliv-300 at ₹61.60)
- Rifaximin: ₹10,147/year savings (Rifaximin-400 from DAVA at ₹16.00 vs. Rifagut-400 at ₹43.80)
- Montelukast: ₹6,110/year savings (Montelukast-10 from JAUSH at ₹1.98 vs. Montek-10 at ₹18.72)
- Febuxostat: ₹5,767/year savings (Febuxostat-40 from JAUSH at ₹2.00 vs. Febuwal-40 at ₹17.80)
- Pantoprazole: ₹5,696/year savings (Pantoprazole-40 from JAUSH at ₹1.21 vs. Pantoziv-40 at ₹16.82)

These annual savings projections assume once-daily consumption and are directly applicable to chronic disease medications requiring long-term daily therapy (e.g., statins, antihypertensives, antidiabetics, proton pump inhibitors, montelukast for asthma prophylaxis). For short-course medications such as antibiotics (Azithromycin, Amoxicillin) and corticosteroids (Prednisolone), per-course rather than annual savings are more clinically relevant.

**FIGURE 8:**
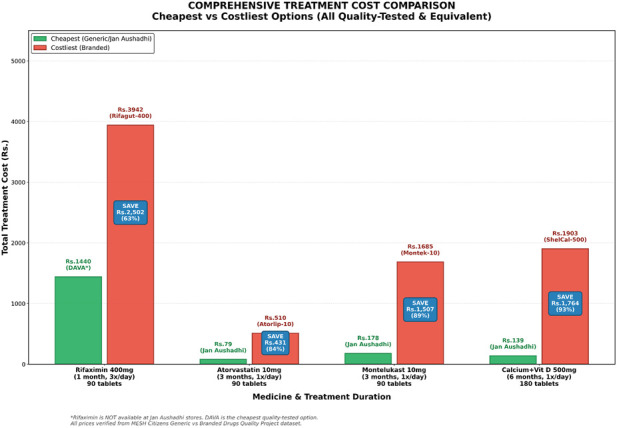
Comprehensive treatment cost comparison – Treatment cost comparison for four common therapeutic regimens, demonstrating potential savings from choosing generic over branded options. All compared products passed quality testing. Rifaximin 400 mg (1-month course, 3×/day, 90 tablets): Cheapest option (DAVA) ₹1,440 vs. costliest (Rifagut-400) ₹3,942; potential savings ₹2,502 (63%). Note: Rifaximin is not available at Jan Aushadhi stores; DAVA represents the cheapest quality-tested option. Atorvastatin 10 mg (3-month course, 1×/day, 90 tablets): Jan Aushadhi ₹79 vs. Atorlip-10 ₹510; potential savings ₹431 (84%). Montelukast 10 mg (3-month course, 1×/day, 90 tablets): Jan Aushadhi ₹178 vs. Montek-10 ₹1,685; potential savings ₹1,507 (89%). Calcium + Vitamin D 500 mg (6-month course, 1×/day, 180 tablets): Jan Aushadhi ₹139 vs. ShelCal-500 ₹1,903; potential savings ₹1,764 (93%). Combined potential savings across these four regimens: ₹6,204. These examples demonstrate that quality-assured generic alternatives can reduce treatment costs by 63%–93% compared to branded products.

When comparing average generic prices to average branded prices, the mean savings was 50% (range: 18%–85%). The highest percentage savings were observed for amlodipine (85%), thyroxine sodium (77%), metformin (74%), and aspirin (73%), while the lowest were for prednisolone (18%), telmisartan (25%), and acetaminophen (29%) (examples are shown in Figure 8).

## Discussion

This study provides a comprehensive evaluation of the pharmaceutical quality and cost of essential medicines available in Kerala, India. Across 131 samples spanning 8 therapeutic categories, generic medicines obtained from government programs, private generic chains, and independent distributors met the pharmaceutical quality standards of the Indian Pharmacopoeia 2022 when assessed against these standards. All samples met specifications for description, uniformity, dissolution, impurities, and assay, indicating that quality differences should not be a limiting factor for the utilization of generic medicines. At the same time, substantial price variation was observed, with generic medicines costing an average of 48.6% less than branded equivalents. Government programs, particularly Jan Aushadhi, consistently offered the lowest-priced options across most therapeutic categories, suggesting significant potential to reduce out-of-pocket expenditure, particularly for patients managing chronic diseases.

A systematic review by McManus and Naughton highlighted that medicine quality sampling studies remain methodologically heterogeneous, with the overall estimated median prevalence of substandard/falsified medicines at 25% - but this figure is heavily influenced by studies from malaria-endemic regions examining the quality of antimalarial drugs (McManus and Naughton, 2020). In contrast, medicines used for non-communicable diseases, which now account for a substantial proportion of disease burden in India, remain comparatively underrepresented in quality assessments. Our findings extend prior work by evaluating a broad range of commonly prescribed medicines and by directly comparing branded and generic products sourced from the same regulated market.

The observed quality equivalence is consistent with earlier studies demonstrating that generic medicines meet established pharmacopeial standards across multiple drug classes. Studies evaluating cardiovascular medicines (Sharma et al., 2024), and antiretroviral therapies (Sapsirisavat et al., 2016). Our study extends these findings across a broader range of therapeutic categories. While reports of substandard medicines in low- and middle-income countries persist (Gnegel et al., 2022), these studies often emphasize the role of weak regulatory oversight and unregulated supply chains. In contrast, our samples were procured from established sources, including government supply programs, recognized pharmaceutical companies, and regulated distribution channels, which likely contributed to the high compliance rates observed.

Despite strong evidence supporting generic equivalence, negative perceptions among healthcare providers and consumers remain a major barrier to uptake. Studies from other countries indicate that generics are often perceived as inferior in quality or safety (Mahdi et al., 2020). Such perceptions can influence prescribing behaviour and limit access to affordable treatment. The present findings provide objective data that may help address these misconceptions, particularly regarding government-supported programs. The inclusion of medicines from the Government’s public medicine scheme, Jan Aushadhi, in this study is particularly relevant in this context. Both central government and state government initiatives, such as Pradhan Mantri Bhartiya Jan Aushadhi Pariyojana (PMBJP) and Kerala Medical Services Corporation Limited (KMSCL), were established to improve access to affordable medicines, yet awareness and trust in the program remain limited in some regions (Chaturvedi et al., 2024). All 21 January Aushadhi samples in this study met pharmacopeial standards, supporting the quality of medicines distributed through this program and reinforcing its role in improving equitable access to medications.

The relationship between the affordability of medicine and health outcomes is well documented. When medicines are unaffordable, patients commonly resort to rationing, dose-splitting, or complete discontinuation, behaviours that increase morbidity, mortality, and paradoxically, long-term healthcare costs. Furthermore, higher out-of-pocket costs are associated with reduced adherence and poorer outcomes across chronic conditions (Dodd et al., 2018; Bansal et al., 2025). By demonstrating that lower-cost generic medicines meet quality standards, this study supports the economic and clinical rationale for generic substitution. These findings are consistent with large systematic reviews and meta-analyses showing no evidence of brand-name superiority across multiple therapeutic classes (Kesselheim, 2011; Kesselheim et al., 2008; Manzoli et al., 2016).

Our findings of 100% quality compliance across all 131 samples align with robust evidence supporting generic-branded equivalence. Kesselheim et al. analysed 47 studies covering nine cardiovascular drug classes and found no evidence of brand-name superiority. Notably, despite this evidence, 53% of editorials expressed negative views toward generic substitution (Kesselheim, 2011). The FDA found average pharmacodynamic differences between generic and brand-name products of approximately 4%, with nearly 98% differing by less than 10% (Kesselheim et al., 2008). Such skepticism has clinical implications: patients prescribed lower-cost generics demonstrate improved medication adherence, whereas “dispense as written” prescriptions are associated with lower fill rates. The most comprehensive meta-analysis by Manzoli et al. of 74 RCTs found that 100% of trials showed non-significant differences between generic and brand-name cardiovascular drugs for soft outcomes and comparable safety profiles (Manzoli et al., 2016).

The quality equivalence we observed supports the theoretical basis for generic substitution. Regulatory requirements mandate that generic medicines demonstrate pharmaceutical equivalence to reference products, and our data suggest these requirements are being met in the Kerala pharmaceutical market. This finding is consistent with quality surveillance data from mature regulatory systems but provides valuable reassurance in the Indian context, where perceptions of generic inferiority persist. Our cost-effectiveness findings are consistent with the economic rationale underlying generic medicine policies. The substantial price differentials we documented represent a significant potential for reducing healthcare costs. This is particularly relevant given evidence that out-of-pocket medication costs adversely affect adherence, with downstream consequences for health outcomes and overall healthcare expenditure.

These findings have several policy implications. First, they support continued investment in and expansion of government generic medicine programs, such as Jan Aushadhi, which provide quality-assured medicines at substantially lower costs. Second, they suggest that prescriber and consumer education regarding generic quality could safely increase utilization, reducing healthcare expenditure without compromising the quality of medicines. Third, they highlight opportunities to implement transparent price-comparison tools at points of prescription and dispensing.

This study utilized Maximum Retail Price (MRP) rather than actual selling price for cost-effectiveness analysis. In India, MRP represents the legally mandated ceiling price inclusive of all taxes under the Legal Metrology Act, 2009 and the Drugs (Prices Control) Order, 2013. This approach provides a standardized, verifiable metric ensuring reproducibility across studies, represents the conservative (maximum) consumer cost estimate, and aligns with regulatory frameworks including National Pharmaceutical Pricing Authority methodology. For government outlets such as Jan Aushadhi Kendras, MRP and selling price are identical. While actual consumer expenditure may be lower than MRP where discounts are available, the savings estimates presented should be interpreted as conservative lower bounds.

Additional limitations should also be acknowledged. The study was geographically limited to Kerala, which has relatively strong healthcare infrastructure; findings may not generalize to other States with weaker regulatory systems. The sample size within individual therapeutic categories was limited, precluding subgroup analyses. The mystery shopping approach may not capture products available through all distribution channels. The 100% pass rate, while reassuring, may reflect selection bias toward legitimate retail outlets; products from unregulated sources might show different quality patterns. Additionally, the study did not employ probability-based sampling of outlets within each source category; the effective sample size for certain comparisons may be smaller than 131 if clustering within manufacturers or batches is substantial. While we documented the manufacturers and batch numbers for each sample, formal cluster-adjusted analyses were not performed given the descriptive nature of the study. The exclusion of products within 3 months of expiry, while necessary to ensure adequate shelf life for testing completion, may reduce detection of stability-related quality failures that could affect products approaching their expiry dates. The samples were obtained at a single time point, and quality may vary across different batches or time periods, and thus, longitudinal surveillance would be needed to assess consistency over time.

This study assessed pharmaceutical equivalence through *in vitro* testing per IP-2022 standards; bioequivalence (*in vivo* pharmacokinetic) testing was outside the study scope. This is appropriate for post-market quality surveillance for several reasons. First, pharmaceutical equivalence through rigorous *in vitro* testing (assay, dissolution, impurities) is the established methodology for quality monitoring of marketed medicines per WHO and IP-2022 guidelines. Second, all products tested were already-marketed formulations that had satisfied regulatory requirements for market authorization, including any necessary bioequivalence documentation at the time of approval. Third, most of the medicines we evaluated (∼46%) were immediate-release formulations of BCS Class I or III drugs, for which *in vitro* dissolution testing is scientifically accepted as predictive of *in vivo* performance under international regulatory guidelines.

Nonetheless, despite these limitations, this study provides valuable evidence regarding the pharmaceutical quality of medicines available in the Indian market. Our findings suggest that the regulatory framework in India is effective in ensuring that marketed medicines, regardless of their branded or generic status, meet established quality standards. The study supports policies promoting the use of generic medicines as a strategy to improve healthcare accessibility while maintaining therapeutic quality. Future studies should expand geographic coverage to states with different regulatory capacities. Longitudinal monitoring could assess quality consistency over time. Inclusion of samples from online pharmacies and informal markets would provide more comprehensive market assessment. Bioequivalence studies, while resource-intensive, would provide definitive evidence of therapeutic equivalence. Finally, implementation research examining strategies to increase appropriate generic utilization despite quality reassurance data would complement quality assessment studies.

To conclude, this quality assessment of medicines from regulated retail outlets in Kerala, India demonstrates that generic medicines from multiple source categories – including government programs, private generic chains, and independent distributors – achieved equivalent pharmaceutical quality to branded products when tested against Indian Pharmacopoeia 2022 standards. The 100% compliance rate across 131 samples representing 22 therapeutic categories provides evidence from this convenience sample that medicines from licensed retail sources in Kerala met pharmacopeial quality standards, offering reassurance regarding generic medicine quality within regulated supply chains. Simultaneously, substantial cost advantages exist for generic medicines, with government programs (particularly Jan Aushadhi) consistently providing quality-assured products at the lowest prices. The potentially significant annual savings by choosing optimal sources represents meaningful economic benefit for patients and healthcare systems. These findings support policies promoting generic medicine utilization and provide evidence for prescriber and consumer education initiatives. Affordable and quality are not mutually exclusive – they can and do coexist in well-regulated pharmaceutical markets. Scaling this model through continued investment in public generic programs, price transparency mechanisms, and quality surveillance can contribute to achieving universal health coverage goals by making essential medicines accessible to all.

Our findings provide supportive evidence for policies promoting generic medicine utilization from regulated sources as a strategy to reduce out-of-pocket healthcare expenditure without compromising therapeutic quality. Healthcare practitioners, policymakers, and consumers may consider this evidence when making decisions that balance clinical efficacy with economic sustainability, while recognizing that broader generalization requires studies across diverse geographic and regulatory settings. The expansion of government generic medicine schemes, combined with targeted education addressing misconceptions about generic quality, represents a potential pathway to improve medicine affordability.

## Data Availability

The datasets presented in this study can be found in online repositories. The names of the repository/repositories and accession number(s) can be found in the article/Supplementary Material.
